# Desmoid Tumor as an Initial Presentation of Familial Adenomatous Polyposis: A Review of the Literature

**DOI:** 10.7759/cureus.2297

**Published:** 2018-03-09

**Authors:** Fady G. Haddad, Sandy El Bitar, Iskandar Barakat, Liliane Deeb

**Affiliations:** 1 Internal Medicine, Staten Island University Hospital, Northwell Health, Staten Island, USA; 2 Department of Hematology/oncology, Staten Island University Hospital, Northwell Health, Staten Island, USA; 3 Gastroenterology, Staten Island University Hospital, Staten Island, USA; 4 Gastroenterology and Hepatology, Staten Island University Hospital, Staten Island, USA

**Keywords:** desmoid tumors, familial adenomatous polyposis, fibromatosis

## Abstract

Desmoid tumors (DTs) are rare soft tissue neoplasms, especially when originating from the small bowel. An association with familial adenomatous polyposis (FAP) of the colon has been well documented. Within the FAP population, there is a strong correlation between prophylactic procto-colectomy and subsequent development of DTs. Very few reports describe cases of FAP initially presenting with desmoid-related complications. Therefore, we underwent a review of the literature in order to delineate the characteristics of desmoid tumors occurring as an initial presentation of FAP.

## Introduction and background

Desmoid tumors or fibromatosis are rare soft tissue neoplasms, especially when originating from the small bowel. They commonly affect the mesenchymal tissues intra-abdominally, within the abdominal wall or in the limb girdle. Their association with familial adenomatous polyposis of the colon (FAP) and Gardner’s syndrome has been well documented. It is reported that abdominal wall and intra-abdominal desmoids occur more frequently in FAP patients, and the incidence ranges from 3.5% to 32%. Within the FAP population, there is a strong correlation between prophylactic procto-colectomy and the subsequent development of desmoid tumors. However, it is rather uncommon for FAP to initially present with desmoid-related complications [1-2].

The term “desmoid” meaning tendon or band in Greek was first used by the German physiologist and comparative anatomist Johannes Müller in 1838. Desmoid tumors (DTs) form only 0.03% of all tumors. Predisposing factors to DT include female gender, prior abdominal trauma or surgery, and family history of fibromatoses. Differential diagnosis include gastrointestinal stromal tumors, lymphoma, and sarcoma. DTs can be primary or secondary. Primary or sporadic DTs are extremely rare and originate from the proliferation of fibroblasts-myofibroblasts with the formation of benign stromal neoplasms. Secondary DTs are commonly associated with hereditary polyposis syndromes with 30% of FAP patients developing intra-abdominal desmoids disease, including tumors, nodules, and sheet-like lesions. They can also occur secondary to trauma or hormonal stimulation [1-2].

## Review

DTs are classified into abdominal wall, extra-abdominal, and intra-abdominal based on their location and clinico-pathological findings. Abdominal wall DTs are proliferative fibrous tumors. Extra-abdominal DTs are histologically similar but more invasive than intra-abdominal DTs. DTs that occur intra-abdominally arise from the mesentery in 85% of cases. Confirmation is based on histo-pathological examination where the findings of spindle- or stellate-shaped fibroblasts in a collagenous stroma favor the diagnosis of DT. Imaging studies such as computed tomography (CT) scan and magnetic resonance imaging (MRI) are often used to delineate the anatomical location and extension of the tumor [3-5].

Small bowel obstruction (SBO) is a common complication of desmoid tumors which is reported in 27% to 58% of patients with FAP-related desmoid tumors in previous studies. However, it is uncommon for FAP to initially present with SBO secondary to DT. In this setting, SBO can be caused either by direct mass effect due to tumor growth, or infiltration of the mesentery by tumor cells causing wrinkling of the bowel margin and sclerosis of the mesenteric vessels which leads to ischemic stricture formation. SBO recurrence is not uncommon and confirms the progressive nature of the disease, as a recent study showed that around two-thirds of patients had one recurrence and up to one-third had a second recurrence. Formation of multiple strictures is often described and attributed to the ability of DT to occur at multiple sites. Other complications of mesenteric DTs include ureteric obstruction, intestinal perforation, entero-cutaneous fistulae, and intestinal hemorrhage [3-5].

Desmoid lesions in FAP are also characterized by the involvement of the mesentery and abdominal wall in the majority of cases, and tumors are often multiple in this setting. Mesenteric DTs present with insidious symptoms that usually occur when the tumor is large enough to cause abdominal discomfort and pain. In addition, mesenteric DTs do not metastasize but they may be locally aggressive. They are similar to fibrosarcoma and fibrous lesions in their biological behavior [6-7].

Initial management includes upper and lower endoscopy to exclude any associated polyposis syndromes. If a polyposis syndrome is found, medical therapy is preferred in view of the high recurrence rate, the practical difficulty, and the risks associated with surgical approaches unless a strict surgical indication is present. First line medications consist of non-steroidal anti-inflammatory drugs (NSAIDs), tamoxifen and other chemotherapeutic agents namely vinblastine, methotrexate, doxorubicin, dacarbazine, and carboplatin. However, surgery is the preferred modality for sporadic DT as recurrence rate is low [6-7].

Prognosis depends on the location of these tumors. Intra-abdominal DTs are associated with unfavorable outcomes because of the increased rate of associated complications like bowel obstruction, bleeding, entero-cutaneous fistulae, and perforation. In cases of retroperitoneal involvement, DTs could result in major blood vessels compression and ureteral obstruction [6-7].

## Conclusions

Desmoid tumors constitute one of the most important extraintestinal manifestations of FAP because they are associated with increased morbidity and mortality. Intra-abdominal desmoid disease represents 30% of cases. CT scan and MRI are used to delineate the anatomical location and extension of the tumor whereas diagnosis is confirmed by pathology and immunohistostaining. Management is challenging and should start with upper and lower endoscopy to exclude underlying polyposis syndromes. Medical therapy is preferred in uncomplicated desmoid disease especially in the setting of FAP. Surgical resection, however, is secluded for sporadic desmoids.
